# Case Report: Associated Ocular Adverse Reactions With Inactivated COVID-19 Vaccine in China

**DOI:** 10.3389/fmed.2021.823346

**Published:** 2022-01-17

**Authors:** Kunpeng Pang, Lijie Pan, Hui Guo, Xinyi Wu

**Affiliations:** Department of Ophthalmology, Qilu Hospital of Shandong University, Jinan, China

**Keywords:** ocular adverse event, vaccine, COVID-19, uveitis, keratitis

## Abstract

The vaccine is still the best clinical measure for effective prevention and control of coronavirus disease 2019 (COVID-19). The vaccine-associated ocular adverse reactions should be noted in detail among the medical community. We reported twelve eyes of 9 patients presented at the Department of Ophthalmology, Qilu Hospital of Shandong University from March to August 2021 with ocular complaints following COVID-19 vaccination. The main inclusion criterion was the development of ocular symptoms within 14 days after receiving a dose of an inactivated COVID-19 vaccine. The mean (SD) age was 44.7 ± 16.5 years (range, 19–78 years), among which seven (77.8%) cases were women. The mean time of ocular adverse events was 7.1 days (range, 1–14 days) after receiving the inactivated COVID-19 vaccine. One patient was diagnosed with choroiditis, 1 with uveitis, 4 with keratitis, 1 with scleritis, 1 with acute retinal necrosis, and 1 with iridocyclitis. Although the causal relationship between vaccines and ocular adverse events cannot be established from this case series report, physicians should pay attention to the ocular adverse reactions following the COVID-19 vaccine administration.

## Introduction

Coronavirus diseases 2019 (COVID-19) is caused by a coronavirus named severe acute respiratory syndrome coronavirus two (SARS-CoV-2). According to the data from WHO, as of November 2021, the COVID-19 pandemic has caused more than 250 million infections and more than 5 million deaths all over the world (1). The vaccine is still the best clinical measure for effective prevention and control of COVID-19 (2). In general, vaccines trigger a protective immune system response against a specified infectious organism through a variety of methods of antigen exposure (3). There are five categories of vaccines including subunits, live-attenuated, inactivated, toxoid, and genetic sequencing vaccines (consisting of DNA or messenger RNA based on genetic sequencing information from the pathogen) (3, 4). So far, more than 292 candidates' vaccines have been being developed for COVID-19 (5).

In China, three types of COVID-19 vaccines have been approved by the Chinese Center for Disease Control and Prevention (CCDC) including inactivated vaccines (Sinopharm, Sinovac, etc.), recombinant vaccine (CHO cells), and adenovirus vector vaccine (Adenovirus Type 5 vector). As of November 2021, more than 2 billion doses of inactivated COVID-19 vaccines have been inoculated in China. We hereby presented a case series of ocular adverse events presenting at Qilu Hospital of Shandong University from March to August 2021, soon after receiving an inactivated COVID-19 vaccine. Color fundus photography was obtained with photography (Carl Zeiss, Germany VISUCAM 224) camera. Optical coherence tomography (OCT) was obtained with a spectral-domain machine (Carl Zeiss, Germany Cirrus HD-OCT 5000). Intraocular pressure was obtained by Topcon, Japan CT•80. Intraocular fluid testing was performed in Beijing GiantMed Diagnostics. Slit-lamp images were taken by Topcon, Japan SL-D7 with optional Digital Photo Attachments. Sex, age, medical history, and clinical data were self-reported and collected.

In this study, twelve eyes of 9 patients presenting with ocular complaints following COVID-19 vaccination were included in this study. The mean (SD) age was 44.7 ± 16.5 years (range, 19–78 years), among which 7 (77.8%) cases were women. The mean time of ocular adverse events was 7.1 days (range, 1–14 days) after receiving the inactivated COVID-19 vaccine. Patients were diagnosed with choroiditis (case 1), uveitis (case 2), keratitis (case 3, 4, 5, 7), scleritis (case 6), acute retinal necrosis (case 8) and iridocyclitis (case 9). The detail of these cases is summarized in Table 1.

**Table 1:** Demographic characteristics of people receiving coronavirus disease 2019 (COVID-19) vaccines and the plans for treatment of these ocular adverse events.

**#**	**Gender**	**Age (years)**	**Ocular history**	**Medical history**	**Diagnosis**	**Vaccine type**	**Days after vaccination**	**Doses**	**Treatment**
1	Female	50	N/A	N/A	Choroiditis (OU)	Inactivated	5	1	One-time periocular triamcinolone acetonide injection; Oral prednisone
2	Female	34	Optic disc vasculitis (OU)	N/A	Uveitis (OU)	Inactivated	5	2	Topical application of prednisolone acetate; Oral prednisone
3	Female	43	N/A	N/A	Keratoconjunctivitis (OD)	Inactivated	7	2	Topical application of ganciclovir ophthalmic gel, tobramycin and dexamethasone
4	Female	55	N/A	N/A	Keratitis (OS)	Inactivated	7	2	Topical application of ganciclovir ophthalmic gel and ciclosporin
5	Female	45	N/A	N/A	Keratitis (OD)	Inactivated	1	1	Topical application of ganciclovir ophthalmic gel and ciclosporin; Intravenous injection of ganciclovir
6	Male	78	Cataract surgery (right eye) for 2 months	10 years history of hypertension	Scleritis (OD)	Inactivated	3	1	Topical application of ganciclovir ophthalmic gel, ciclosporin, tobramycin and dexamethasone; Oral aciclovir tablets
7	Male	19	N/A	N/A	Keratitis (OU)	Inactivated	14	2	Topical application of ganciclovir ophthalmic gel and ciclosporin; Intravenous injection of ganciclovir
8	Female	46	N/A	N/A	Acute Retinal Necrosis (OD)	Inactivated	8	N/A	Intravenous and intravitreous injection of ganciclovir; Oral prednisone
9	Female	32	Recurrent iritis (both eyes) for 6 years	N/A	Iridocyclitis (OS)	Inactivated	14	3	Topical application of prednisolone acetate, tobramycin and dexamethasone

## Selected Cases Description

### Case 2

**Figure 1:**
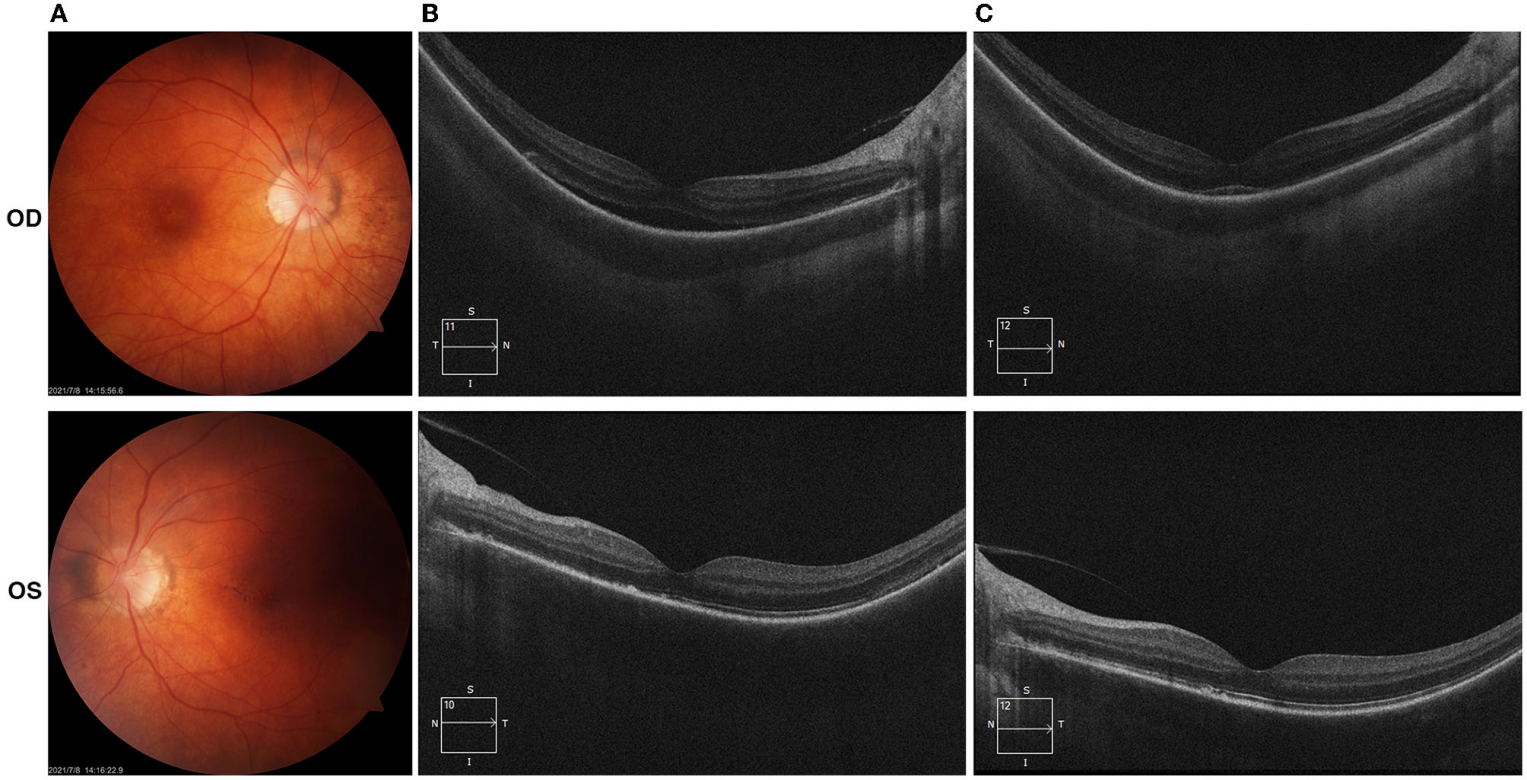
(Case 2) Color fundus and optical coherence tomography (OCT) examinations. **(A)** Fundus examinations at the presentation in May 2021(2 months after the vaccination); **(B)** OCT photos in May 2021 demonstrated the foveal neurosensory detachment in the right eye; **(C)** OCT photos in July 2021, most of the retinal neurosensory layer in foveola back to the retinal pigment epithelium with the hyperreflective subfoveal lesion.

Patient 2 had an ocular history of optic disc vasculitis in her both eyes 1 month after she received the flu shot in November 2020 and was resolved after receiving a tapering dose of oral prednisone. She denied having other medical histories. In March 2021, she presented to our clinic with blurred vision in both eyes 5 days after the administration of the second dose of inactivated COVID-19 vaccine. The best-corrected visual acuity (BCVA) was 20/25 in both eyes. Fine dust-like keratic precipitates (KP) and flare in the anterior chamber were noticed in both eyes. Two small iris nodules were seen at the pupillary margin in her left eye. Pigment deposits were dispersed on the surface of the lens. The edge of both eyes' optic discs was unclear. The patient received the topical application of prednisolone acetate and oral prednisone 5 mg per day. In May 2021, the patient complained about vision loss and visual distortions in her both eyes. The dilated fundus examination (OD/OS) reveals signs of “sunset glow” (Figure 1A). The foveal neurosensory detachment in the right eye was noticed by OCT (Figure 1B). In addition to the topical application of prednisolone acetate, the patient received a systemic tapering dose of glucocorticoids and ciclosporin. At 2-month follow-up, the BCVA in both eyes was back to 20/20. Most of the retinal neurosensory layer in foveola returned back to the retinal pigment epithelium in the right eye (Figure 1C).

### Case 4

Patient 4 complained about eye redness and blurred vision in her left eye 7 days after receiving the second dose of inactivated COVID-19 vaccine in May 2021. She denied the medical history. In August 2021, she presented to our clinic with seriously blurred vision in her left eye. The BCVA was 20/20 in her right eye and 20/200 in her left eye. The intraocular pressure(IOP) was 16 mmHg in the right eye and 15 mmHg in the left eye. Slit-lamp examination revealed a dendritic ulcer with terminal bulbs in the central cornea and it becomes readily apparent with fluorescein staining (Supplementary Figure 1A). Ganciclovir gel and cyclosporine eyedrop was used for 4 weeks. The cornea ulcer healed with a light nebula left and the BCVA of her left eye returned to 20/100 (Supplementary Figure 1B).

### Case 5

**Figure 2:**
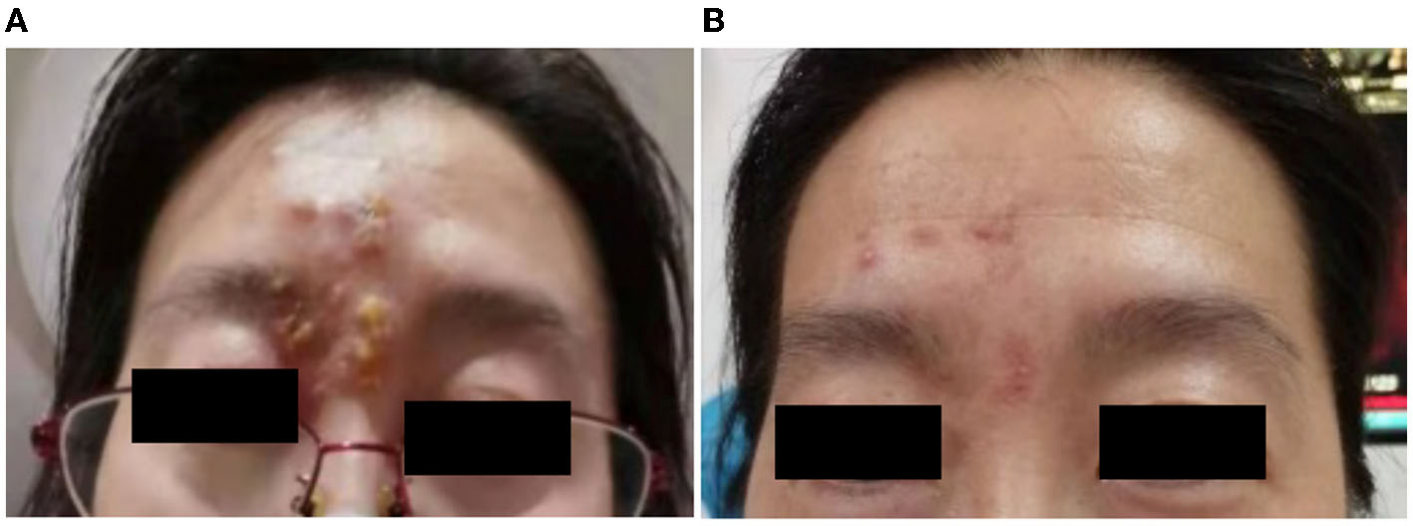
(Case 5) Facial blisters photos. **(A)** 1 day after receiving the first dose of coronavirus disease 2019 (COVID-19) vaccine; **(B)** 2 months after the vaccine injection. After receiving an intravenous injection of ganciclovir, the blisters healed gradually with remained scabs on her right forehead **(B)**.

Patient 5 presented to our clinic in June 2021 with foreign body sensation, lacrimation, and redness in her red-eye, accompanied by groups of fluid-filled blisters on her right forehead 1 day after receiving the first dose of vaccine (Figure 2A). The BCVA at the presentation was 20/20 in her right eye and 20/25 in her left eye. The intraocular pressure was 17 and 16 mmHg, respectively. The patient had edema in her right eyelid, conjunctival congestion, shallow corneal ulcer close to the limbus, and positive fluorescein staining in her right eye. No cells and flare were noticed in the right eye anterior chamber. After being given an intravenous injection of ganciclovir and plus with topical application of ganciclovir gel and cyclosporine eyedrop for 2 months, the facial blisters and corneal ulcer in her right eye healed (Figure 2B, Supplementary Figure 2).

### Case 6

**Figure 3:**
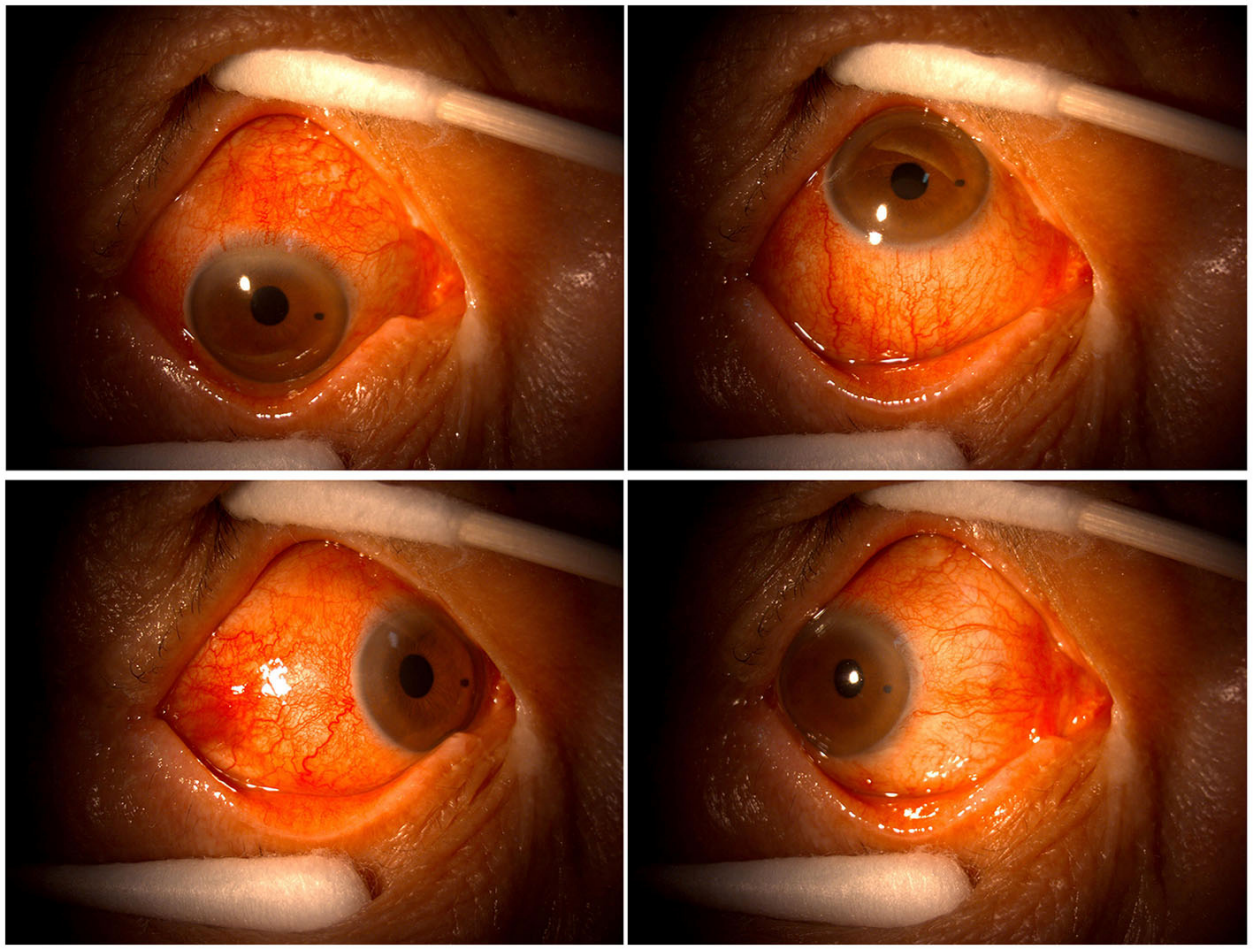
(Case 6) Slit-lamp examination photos at the presentation.

Patient 6 had a 10-year medical history of hypertension. Before presenting to our clinic in August 2021, he had accepted a cataract surgery in his right eye for 2 months. Three days after receiving the first dose of COVID-19 inactivated vaccine in July 2021, he complained the redness, lacrimation, and visual loss in his right eye, and was diagnosed as having scleritis in a local clinic. He was given the treatment of the eyedrops of tobramycin and dexamethasone for 1 month in his right eye, and the symptoms were getting worse. In August, he was referred to our clinic. The BCVA at the presentation was 20/50 OD and 20/67 OS. The intraocular pressure was 19 and 20 mmHg, respectively. The slit lamp examinations revealed serious conjunctival congestion and a 3 mm × 3 mm nodule on the temporal sclera of his right eye. The cornea was clear, and no cells or flares were observed in the anterior chamber (Figure 3).

## Discussion

Since the role of vaccination is to prevent diseases, the vaccine has been widely administered to a large group of healthy individuals. The safety and side effects raise concerns among the human community. Local reactions (swelling, erythema, and pain around the site of injection) at injection sites and mild systemic reactions (transient fever, tiredness, headache, or chills) are common, but serious side effects are uncommon. According to the data from CCDC (From December 2020 to April 2021), the adverse events of COVID-19 vaccines were reported at 11.86 per 100,000 persons in China (6). Regarding this, 82.96% of the total events were general reactions including fever, swollen and callosity at injection sites, and 17.04% were uncommon adverse reactions such as allergic skin rash, angioedema, and acute severe allergic reaction.

Here, we reported 9 cases of ocular adverse events after receiving inactivated COVID-19 vaccines, although the causal relationship cannot be established in this study. Cases 1, 2, and 9 (5 of 6 eyes) were different types of uveitis and were reported at a mean of 8 days after vaccination. After being given the topical application and oral steroids, all their clinical manifestations disappeared and BCVA was back to a normal level. Cases of uveitis in association with vaccine administration have been reported with nearly all vaccines (7). Benage et al. reported a total of 289 cases of vaccine-associated uveitis between 1984 and 2014, and the median time from vaccination to uveitis onset was 16 days (7), among which 199 cases occurred in women vs. 77 in men. Our reports were consistent with their findings that all three cases were women. The literature of COVID-19 vaccines-related uveitis was rare. Renisi et al. reported a case of anterior uveitis 14 days after the second dose of BNT162b2 COVID (8). The precise pathogenesis frequently remains unclear. Different mechanisms have been hypothesized including the direct infection by the attenuated but still, active virus strain and inflammation induced by one or more adjuvants (such as aluminum salts), routinely used in inactivated or subunit/conjugate vaccines (9). Adjuvants are used to enhance the immunogenicity response and result in a reduced frequency and amount of vaccination required to obtain adequate preventive immunity (10). Adaptive and the innate arms of the immune system are both influenced by adjuvants via various mechanisms including the activation of Toll-like receptors, NOD-like receptors, etc., and lead to their downstream cytokines generation. Moreover, the activation of antigen presented cells transfers the antigen to B-cell and T-cell leading to the heightened adaptive immune response to antigen (11–13). The autoinflammation and autoimmune conditions induced by adjuvants, known as Shoenfeld syndrome, often occur in patients with a personal or history of autoimmune disease (9, 14). However, the presented patients denied medical history or family history of autoimmune disease. The enhancement of immune response might play an important role in these uveitis cases.

In addition to uveitis, keratitis was reported in 4 of 9 cases (age range between 19 and 55 years) at a mean of 14 days (range between 1 and 43 days) after the administration of the vaccine. Although we did not do the PCR for virus test to confirm the type of keratitis, all cases were resolved with topical application of ganciclovir ophthalmic gel and ciclosporin or systemic ganciclovir. Keratitis in association with vaccines appeared to be uncommon. Furthermore, three cases of herpes simplex virus (HSV) keratitis were reactivated after COVID-19 vaccination (2 of 3 were associated with BNT162b2 mRNA vaccination, the third one was AstraZeneca) (15, 16). A review reported 24 cases of keratitis in association with herpes zoster or varicella vaccination (17). Two possible mechanisms involved in the pathogenesis included molecular mimicry and autoinflammation triggering the host response and promoting the HSV replication (16). Hence, the treating physician should be mindful of such associations between vaccination and keratitis.

The vaccine-associated scleritis was uncommon. Pichi et al. (18) reported a case of episcleritis and 2 cases of anterior scleritis which were noted soon after the administration of inactivated COVID-19 vaccines. Consistent with the report, the presented case of scleritis was noted 3 days after receiving the first dose of the vaccine. The symptoms of the case were mild and responsive to glucocorticoids. Noteworthily, there were two reports of scleritis (one case of episcleritis and two cases of anterior scleritis) after the outbreak of coronavirus in 2019 (19, 20).

A case of varicella-zoster virus (VZV) related acute retinal necrosis (ARN) was reported previously following one dose of the COVID-19 vaccine (21). Case 8 was presented to our clinic 8 days after the vaccination. PCR from the aqueous humor sample was positive for both HSV and VZV. Of note, 4 cases of ARN (3 cases were HSV related, 1 case was VZV related) have been reported after the infection of SARS-CoV-2 (22–24). The unconceivable risk of aberrant immune reactions leading to the reactivation of HSV or VZV needs to be kept in mind after the COVID-19 vaccination.

## Conclusion

Although we reported 9 cases of COVID-19 vaccine-related ocular adverse events, the causal relationship cannot be established in this study design. Due to the serious complications of coronavirus diseases 2019, vaccination is still the most effective way to combat the SARS-CoV-2 at present. The benefits of immunization against the virus far outweigh the risks. However, physicians should keep an eye on the ocular adverse reactions following the COVID-19 administration.

## Data Availability Statement

The original contributions presented in the study are included in the article/Supplementary Material, further inquiries can be directed to the corresponding author/s.

## Ethics Statement

The studies involving human participants were reviewed and approved by Medical Ethics Committee of Qilu Hospital of Shandong University. The patients/participants provided their written informed consent to participate in this study. Written informed consent was obtained from the individual(s) for the publication of any potentially identifiable images or data included in this article.

## Author Contributions

KP, LP, HG, and XW were all the treating physicians of the patient. KP and LP recollected all the medical history of the patient and wrote the core of this article. HG and XW helped in the reviewing and editing of the article. All authors contributed to the article and approved the submitted version.

## Funding

This study was funded by National Natural Science Foundation of China (81770893).

## Conflict of Interest

The authors declare that the research was conducted in the absence of any commercial or financial relationships that could be construed as a potential conflict of interest.

## Publisher's Note

All claims expressed in this article are solely those of the authors and do not necessarily represent those of their affiliated organizations, or those of the publisher, the editors and the reviewers. Any product that may be evaluated in this article, or claim that may be made by its manufacturer, is not guaranteed or endorsed by the publisher.

## Supplementary Material

Supplementary Figure 1 (Case 4) Slit-lamp images after receiving the second dose of COVID-19 inactivated vaccine. **(A)** photos taken in August 2021; **(B)** photos taken in September 2021.

Supplementary Figure 2 (Case 5) Slit-lamp examinations of the cornea. **(A)** 2 months after receiving the first dose of COVID-19 vaccine; **(B)** 2.5 months after the vaccine injection. Unfortunately, we missed the first day's slit lamp photos of the cornea. The corneal ulcer healed with slight nebula **(B)** after receiving intravenous injection of ganciclovir and plus with topical application of ganciclovir gel and cyclosporine eyedrops.
