# Supplementary figures and images for: Case Report: Associated Ocular Adverse Reactions With Inactivated COVID-19 Vaccine in China
Source: Front Med (Lausanne). 2022 Jan 17;8:823346. doi: 10.3389/fmed.2021.823346 (PMC8801805; doi:10.3389/fmed.2021.823346)

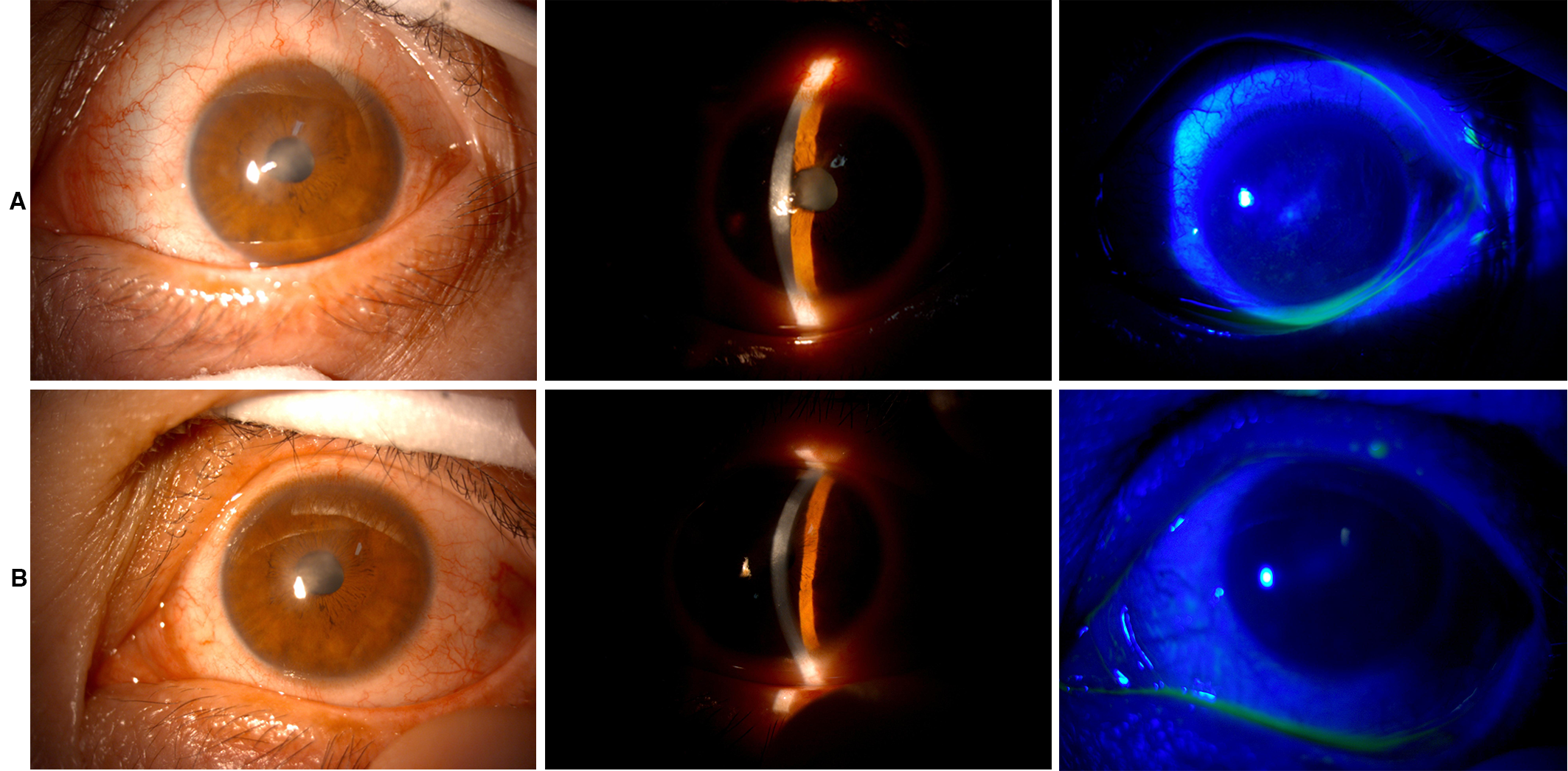

Supplement: Supplementary Figure 1 — (Case 4) Slit-lamp images after receiving the second dose of COVID-19 inactivated vaccine. (A) photos taken in August 2021; (B) photos taken in September 2021. [file Image_1.TIF]

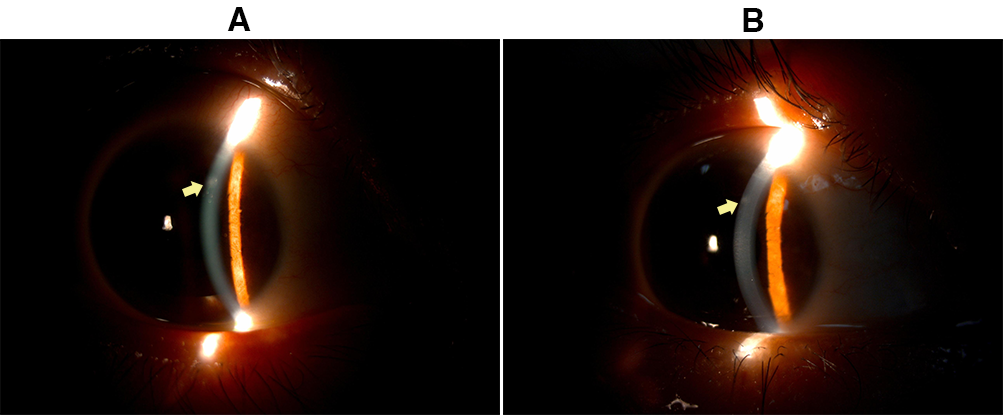

Supplement: Supplementary Figure 2 — (Case 5) Slit-lamp examinations of the cornea. (A) 2 months after receiving the first dose of COVID-19 vaccine; (B) 2.5 months after the vaccine injection. Unfortunately, we missed the first day's slit lamp photos of the cornea. The corneal ulcer healed with slight nebula (B) after receiving intravenous injection of ganciclovir and plus with topical application of ganciclovir gel and cyclosporine eyedrops. [file Image_2.TIF]
